# Supplementary material for: Genome‑wide identification and characterization of miR396 family members and their target genes GRF in sorghum (Sorghum bicolor (L.) moench)
Source: PLoS One. 2023 May 10;18(5):e0285494. doi: 10.1371/journal.pone.0285494 (PMC10171670; doi:10.1371/journal.pone.0285494)
Supplement: S1 Table — (DOCX) [file pone.0285494.s001.docx]

**S1 Table. Primers of qRT-PCR.**

| Gene name | Primer | Primer sequence（5'-3'） |
| --- | --- | --- |
| *Sbi-miR396a* | RT-primer | GTCGTATCCAGTGCAGGGTCCGAGGTATTCGCACTGGATACGACCAGTTC |
|  | 5' primer | CGCGTTCCACAGCTTCTT |
| *Sbi-miR396b* | RT-primer | GTCGTATCCAGTGCAGGGTCCGAGGTATTCGCACTGGATACGACCAGTTC |
|  | 5' primer | CGCGTTCCACAGCTTCTT |
| *Sbi-miR396c* | RT-primer | GTCGTATCCAGTGCAGGGTCCGAGGTATTCGCACTGGATACGACAAGTTC |
|  | 5' primer | CGCGTTCCACAGCTTTCTT |
| *Sbi-miR396d* | RT-primer | GTCGTATCCAGTGCAGGGTCCGAGGTATTCGCACTGGATACGACCAGTTC |
|  | 5' primer | CGCGCTCCACAGGCTTTCTT |
| *Sbi-miR396e* | RT-primer | GTCGTATCCAGTGCAGGGTCCGAGGTATTCGCACTGGATACGACCAGTTC |
|  | 5' primer | CGCGTTCCACAGGCTTTCTT |
| *U6* | RT-primer | GTCGTATCCAGTGCAGGGTCCGAGGTATTCGCACTGGATACGACTGCTAA |
|  | 5' primer | GCGTTGGAACGATACAGAGAAGA |
| *SbiGAPDH* | Forward | CAACGAGTGGGGATACAGCA |
|  | Reverse | TCCGGGGAAGAAGACAACTC |
| *SbiGRF1* | Forward | TACGGCGTTGATCTGGGAGC |
|  | Reverse | CACCATTACGGTAGCGGGAG |
| *SbiGRF2* | Forward | CACTGCGGCGTTTCTTCG |
|  | Reverse | GGCTCACCGTTGTGGTATCG |
| *SbiGRF3* | Forward | CCAAACCACCACATTTCAAGC |
|  | Reverse | CTGCTTGGCGGTCACATAGTC |
| *SbiGRF4* | Forward | CATCTGTTGCCTTCTGATTTCCT |
|  | Reverse | CAGCCGCTTGGTTTGGTAC |
| *SbiGRF5* | Forward | GCTGGCTCATCGCATCTCA |
|  | Reverse | GGCCAGAATCTCTGCCCTTAG |
| *SbiGRF6* | Forward | TCTGCTCCAATGGCATCCTC |
|  | Reverse | GTTCTTGTTCAAAACCTCCCC |
| *SbiGRF8* | Forward | ACTTCTCAGCGTCACATTCTTCC |
|  | Reverse | GGTCCATCATCAATTGTTTCCC |
| *SbiGRF9* | Forward | ACCAACCTAAGAAGACCACCAA |
|  | Reverse | GTTTGTTTGCTTTCTCTCTGTTCA |
| *SbiGRF10* | Forward | GCTTTTACTCCGACGACCACT |
|  | Reverse | ACAGCAGCGGTGTCAGAGG |
