# Supplementary material for: Genome‑wide identification and characterization of miR396 family members and their target genes GRF in sorghum (Sorghum bicolor (L.) moench)
Source: PLoS One. 2023 May 10;18(5):e0285494. doi: 10.1371/journal.pone.0285494 (PMC10171670; doi:10.1371/journal.pone.0285494)
Supplement: Attachment — Submitted filename: Comments.doc [file pone.0285494.s002.doc]

**“Genome‑wide identification and characterization of miR396 family members and their target genes GRF in sorghum (Sorghum bicolor (L.) moench)” is a massive piece of work. I thank the authors for this work. Some comments/suggestions are given below point-wise that will help in improving the text and better presentation of the manuscript.**

1. The quality of the images is very low and difficult to read and understand
2. Why did you investigate the secondary structure of miRNAs? What useful information does it provide for the purpose of this manuscript?
3. In figure 6, the clusters I to V are not specified. Therefore, the results written in the Phylogeny and classification of SbiGRFs section cannot be understood well. Which part of the dendrogram is cluster V?
4. In my opinion, it would be better to study based on GRFs (not miRNAs), and the analysis of GRFs-targeted miRNAs should be only one part of the manuscript because the information of miRNAs is not very useful but analyses such as duplication and selection pressure, exon-intron structure, promoter analysis, transcript-targeted miRNA and simple sequence repeat markers prediction, codon usage bias analysis, and three-dimensional structure of GRFs provide more information to the reader and it is easier to use the information for the desired genetic changes.

**Taking the above into consideration, I recommend this manuscript for publication after applying the changes.**
