# Supplementary material for: Genome‑wide identification and characterization of miR396 family members and their target genes GRF in sorghum (Sorghum bicolor (L.) moench)
Source: PLoS One. 2023 May 10;18(5):e0285494. doi: 10.1371/journal.pone.0285494 (PMC10171670; doi:10.1371/journal.pone.0285494)
Supplement: Attachment — Submitted filename: Response to Reviewers.docx [file pone.0285494.s003.docx]

Dear Editor and Reviewers,

We are very grateful for your critical comments and suggestions. According to these comments and suggestions, we have made revisions on the manuscript. We accepted all the suggestions of editor and reviewers. Our point-by-point responses to the editor and reviewers’ comments are as follows.

**Editor:**

**1-Please amend your authorship list in your manuscript file to include author Dr. Jingxue Wang.**

***Answer:*** The author Dr. Jingxue Wang was added as follows:

Huiyan Wang^1,2^, Yizhong Zhang^1,2*^, Du Liang^1,2^, Xiaojuan Zhang^1,2^, Xinqi Fan^1,2^, Qi Guo^1,2^, Linfang Wang^1,2^, Jingxue Wang^3^, Qingshan Liu^2*^

^3^ School of Life Science, Shanxi University, Taiyuan 030031, Shanxi Province, China

**Reviewer 1:**

**1-The quality of the images is very low and difficult to read and understand**

***Answer:*** All images had been modified.

**2-Why did you investigate the secondary structure of miRNAs? What useful information does it provide for the purpose of this manuscript?**

***Answer:*** The stable secondary stem–loop structure and its conservation are the judgment criteria for miRNA generation. Thus, the secondary structure of miRNAs must be investigated.

**3-In figure 6, the clusters I to V are not specified. Therefore, the results written in the Phylogeny and classification of SbiGRFs section cannot be understood well. Which part of the dendrogram is cluster V?**

***Answer:*** The clusters I to V had been specified. The figure is as follows:

**4-In my opinion, it would be better to study based on GRFs (not miRNAs), and the analysis of GRFs-targeted miRNAs should be only one part of the manuscript because the information of miRNAs is not very useful but analyses such as duplication and selection pressure, exon-intron structure, promoter analysis, transcript-targeted miRNA and simple sequence repeat markers prediction, codon usage bias analysis, and three-dimensional structure of GRFs provide more information to the reader and it is easier to use the information for the desired genetic changes.**

***Answer:*** In plants, miRNA bind to the complementary sites within target mRNA to repress gene expression at the posttranscriptional level, thereby regulating plant growth, development, and response to adverse stress. Currently, studies of plant miRNA mainly focus on a few model plants such as *Arabidopsis* and rice, which are less studied in coarse cereals. This study aimed to investigate how sorghum *miRNA396* is involved in regulating its growth and development. Therefore, all studies were based on *miRNA396*, including its target gene GRF.

**Reviewer 2:**

**1- The abstract must be rewritten with more detailed information in terms of results**

***Answer:*** We have rewritten the abstract. See the section “Abstract” at line 23-46 of page 1:

MicroRNAs (miRNAs) widely participate in plant growth and development. The *miR396* family, one of the most conserved miRNA families, remains poorly understood in sorghum. To reveal the evolution and expression pattern of *Sbi-miR396* gene family in sorghum, bioinformatics analysis and target gene prediction were performed on the sequences of the *Sbi-miR396* gene family members. The results showed that five *Sbi-miR396* members, located on chromosomes 4, 6, and 10, were identified at the whole-genome level. The secondary structure analysis showed that the precursor sequences of all five *Sbi-miR396* potentially form a stable secondary stem–loop structure, and the mature miRNA sequences were generated on the 5′ arm of the precursors. Sequence analysis identified the mature sequences of the five *sbi-miR396* genes were high identity, with differences only at the 1st, 9th and 21st bases at the 5' end. Phylogenetic analysis revealed that *Sbi-miR396a*, *Sbi-miR396b*, and *Sbi-miR396c* were clustered into Group I, and *Sbi-miR396d* and *Sbi-miR396e* were clustered into Group II, and all five *sbi-miR396* genes were closely related to those of maize and foxtail millet. Expression analysis of different tissue found that *Sbi-miR396d*/*e* and *Sbi-miR396a*/*b*/*c* were preferentially and barely expressed, respectively, in leaves, flowers, and panicles. Target gene prediction indicates that the growth-regulating factor family members (*SbiGRF1*/*2*/*3*/*4*/*5*/*6*/*7*/*8*/*10*) were target genes of *Sbi-miR396d*/*e*. Thus, *Sbi-miR396d*/*e* may affect the growth and development of sorghum by targeting *SbiGRF*s. In addition, expression analysis of different tissues and developmental stages found that all *Sbi-miR396* target genes, *SbiGRF*s, were barely expressed in leaves, root and shoot, but were predominantly expressed in inflorescence and seed development stage, especially *SbiGRF1*/*5*/*8*. Therefore, inhibition the expression of *sbi-miR396d*/e may increase the expression of *SbiGRF1*/*5*/*8*, thereby affecting floral organ and seed development in sorghum. These findings provide the basis for studying the expression of the *Sbi-mir396* family members and the function of their target genes.

**2- Authors are using RNA-seq data to describe tissue specific expression levels of these genes. In my opinion, authors should select some sorghum miR396 and GRF genes and perform qRT-PCR analysis to testify the results obtained from RNA-seq database.**

***Answer:*** It had been corrected. See the section “Expression analysis of *SbiGRF*s in different tissues and developmental stages” at line 316-325 of page 16:

To further clarify the role of *Sbi-miR396* and their target gene *SbiGRF*s in inflorescence and seed development, qRT-PCR was performed from young spikes of the inflorescence developmental stage and early development seeds, and the results are shown in Fig 10. Consistent with the RNA-seq results, *Sbi-miR396a*, *Sbi-miR396b*, and *Sbi-miR396c* were barely expressed in the young spikes of the inflorescence developmental stage and early development seeds, while *Sbi-miR396d* and *Sbi-miR396e* were preferentially expressed (Fig 10A). All *Sbi-miR396* target genes, *SbiGRF*s, were expressed at both developmental stages, and were expressed slightly higher in the inflorescence development stage than in the seed development stage. Furthermore, *SbiGRF1*/*5*/*8* was predominantly expressed at both developmental stages, while other *SbiGRFs* showed slightly lower expression levels (Fig 10B).

**3- In M&M section the RNA-seq methodology has be described clearly**

***Answer:*** It had been corrected. See the section “RNA isolation and qRT-PCR analysis” at line 155-177 of page 7-8:

RNA isolation and qRT-PCR analysis

Three materials were used for qRT-PCR analysis, namely, 10480A, L17R and Jinnuo 3. Total RNA was extracted from young spikes of the inflorescence developmental stage and early development seeds using the RNAprep Pure Plant Kit (TIANGEN, Beijing, China) following the manufacturer’s instructions. The purity and content of total RNA were detected by 1.0% agarose gel electrophoresis and NanoDrop 2000 (Thermo Fisher Scientific，USA). First-strand cDNA of miRNA and mRNA were synthesized with the Mir-X miRNA First-Strand Synthesis Kit and the PrimeScript^TM^ RT reagent Kit with gDNA Eraser (Perfect Real Time) (Takara, Beijing, China), respectively. Quantitative real-time PCR (qRT-PCR) of *Sbi-miR396* and *SbiGRF*s were performed with three technical and three biological replicates in the LightCyclerTM 96 (Roche, Basel) using the Mir-X miRNA qRT-PCR TB Green Kit and the TB Green *Premix Ex Taq*^TM^ II (Tli RNaseH Plus) (Takara, Beijing, China), respectively. The *U6* and *SbiGAPDH* gene were used as internal reference. The qRT-PCR reaction system of *Sbi-mi396* contained 10 μL 2×TB Green Advantage Premix, 0.4 μL (10 μM) RT-primer, 0.4 μL (10 μM) 5' primer (S1 Table), 2 μL cDNA template, 0.4 μL 50× Rox Reference Dye II, and 6.8 μL ddH_2_O. The reaction procedure was as follows: denaturation at 95 ℃ for 10 s, followed by 40 cycles at 95 ℃ for 5 s and 60 ℃ for 20 s. The qRT-PCR reaction system of *SbiGRF*s contained 10 μL 2× TB Green *Premix Ex Taq*, 0.8 μL (10 μM) forward primer, 0.8 μL (10 μM) reverse primer (S1 Table), 2 μL cDNA template, 0.4 μL 50× Rox Reference Dye II, and 6.0 μL ddH_2_O. The reaction procedure was as follows: denaturation at 95 ℃ for 3 min, followed by 40 cycles at 95 ℃ for 10 s and 60 ℃ for 30 s. After the reaction was completed, relative expression levels were calculated using the△CT method. Statistical significance was calculated using SPSS 19.0 software (SPSS Corp., Chicago, IL).

**4- How were the gene expression count tables normalized, and what tool did you use?**

***Answer:*** The *U6* and *SbiGAPDH* gene were used as the internal reference to normalize the gene expression data in qRT-PCR of *Sbi-miR396* and *SbiGRF*s. This information is described in the section “RNA isolation and qRT-PCR analysis” at line 166-177 of page 8.

**5- The quality of figures should be increased significantly.**

***Answer:*** All images have been modified.

**6- For the evolution study it is necessary to add more species (algae, moss, fern, some outgroup).**

***Answer:*** Algae, moss, and fern do not have miR396.
