# Supplementary material for: Genome‑wide identification and characterization of miR396 family members and their target genes GRF in sorghum (Sorghum bicolor (L.) moench)
Source: PLoS One. 2023 May 10;18(5):e0285494. doi: 10.1371/journal.pone.0285494 (PMC10171670; doi:10.1371/journal.pone.0285494)
Supplement: Attachment — Submitted filename: Response to Reviewers.docx [file pone.0285494.s004.docx]

Dear Editor,

We have made revisions on the manuscript following your comments and suggestions. All changes made in the manuscript are in red. Our point-by-point responses to the editor’ comments are as follows.

**Reviewer 2:**

**1- The statistical analysis conducted to examine the expression pattern of the selected genes lacked clarity, making it difficult to determine which gene expression changes were significant. Please revise the qRT-PCR results and Figure 10 accordingly.**

***Answer:*** qRT-PCR results and Figure 10 had been revised. See the qRT-PCR results at line 320-331 of page 16:

*Sbi-miR396a*, *Sbi-miR396b*, and *Sbi-miR396c* were expressed at low levels or not expressed in the young spikes of the inflorescence developmental stage and early development seeds, while *Sbi-miR396d* and *Sbi-miR396e* were preferentially expressed (Fig 10A). Moreover, *Sbi-miR396d* was expressed slightly higher than *Sbi-miR396e* at both developmental stages. Nine *Sbi-miR396* target genes, *SbiGRF*s, were expressed at both developmental stages, and most *SbiGRF*s were expressed slightly higher in the inflorescence development stage than in the seed development stage (Fig 10B). In sorghum varieties, *SbiGRF8* exhibited the highest expression level, followed by *SbiGRF1* and *SbiGRF5*. *SbiGRF9* and *SbiGRF4* showed the lowest expression level. *SbiGRF2*, *SbiGRF3*, *SbiGRF6*, and *SbiGRF10* showed intermediate expression level. Furthermore, *SbiGRF8* and *SbiGRF1* were expressed at about two times that of *SbiGRF5* and about ten times that of *SbiGRF9* and *SbiGRF4*. These findings suggest that *Sbi-miR396d*/*e* and *SbiGRF8*/*1*/*5* play a major role in inflorescence and seed development stage in sorghum.

The figure is as follows:

**2- Although qRT-PCR analysis was performed and its findings were presented, they were not discussed section of the article. Please address this in the manuscript.**

***Answer:*** It had been added.

See the section “Discussion” at line 368-379 of page 18:

The phylogenetic evolution analysis showed that *Sbi-miR396d* and *Sbi-miR396e* and *Osa-miR396e* and *Osa-miR396f* were clustered in the same group (Group II), so we speculated that they may have similar functions. Zhang et al. [39] reported that *Osa-miR396f* double mutant significantly increased panicle number, grain length, grain width, and yield compared with WT. In this study, qRT-PCR results showed *Sbi-miR396a*, *Sbi-miR396b*, and *Sbi-miR396c* were expressed at low levels or not expressed in the young spikes of the inflorescence developmental stage and early development seeds, while *Sbi-miR396d* and *Sbi-miR396e* were highly expressed at both developmental stages. These results suggest that only *Sbi-miR396d* and *Sbi-miR396e* are extensively involved in the growth and development of organs and tissues, especially the panicle development in sorghum. Therefore, it is possible to change the panicle and grain development and increase the yield by regulating the expression level of *Sbi-miR396d* and *Sbi-miR396e* in sorghum.

See the section “Discussion” at line 400-425 of page 19-20:

The phylogenetic analysis demonstrated that *SbiGRF8*/*6*/*9* were clustered with *OsGRF6*/*7*/*8*/*9* in Group I, highlighting their close phylogenetic relationship. Research in rice has demonstrated that *OsGRF6* plays a vital role in regulating plant height, stem elongation, and floral organ development[31, 35]; *OsGRF7* mainly controls plant height, leaf length, and tiller number[13, 29]; *OsGRF8* regulates grain size and yield[33]; and *OsGRF9* is not involved in rice growth and development regulation but regulates rice blast resistance[48]. Regarding expression analysis of *SbiGRF*s in different tissues and developmental stages, the expression level of *SbiGRF8* was higher than that of other *SbiGRF*s in the young spikes of the inflorescence developmental stage and early development seeds. Thus, *SbiGRF8* may play a major role in regulating the development of floral organs and seeds in sorghum. *SbiGRF9* exhibited the lowest expression level at both developmental stages, suggesting that this gene is not associated with the regulation of sorghum growth and development. It may be functionally consistent with the homologous gene *OsGRF9* in rice and plays a role in disease resistance. *SbiGRF5*/*3*/*4* were clustered with *OsGRF3*/*4*/*5* in Group V. In rice, *OsGRF3*/*4* regulate grain size[21, 32, 34], and *OsGRF5* regulates plant height and inflorescence development [49]. In the present study, *SbiGRF5* were found to be highly expressed in inflorescence developmental and early development seeds of sorghum. In particular, *SbiGRF5* was preferentially expressed after inflorescences emergence, whereas *SbiGRF3*/*4* showed relatively low expression levels in various tissues. These results suggest that in sorghum, *SbiGRF5* was associated with the regulation of seed development and floral organ development. Furthermore, *SbiGRF2* and *SbiGRF10* also showed relatively low expression levels, whereas *SbiGRF1* exhibited very high expression levels at both developmental stages. These results imply that *SbiGRF*s may be both redundant and functionally specialized in regulating the growth and development of sorghum flower organs and seeds. Therefore, increasing the expression level of *SbiGRF*s, especially *SbiGRF8*, *SbiGRF1* and *SbiGRF5*, by means of genetic engineering may affect the panicle and grain development, and increase the yield in sorghum.
